# Editorial: The role of epigenetic modification in radiotherapy and immuno-oncology in cancer: from bench to clinical application

**DOI:** 10.3389/fonc.2023.1213513

**Published:** 2023-05-16

**Authors:** Hon-Yi Lin, Michael W. Y. Chan, Vivian W. Y. Lui, Peter J. K. Kuppen, Shih-Kai Hung

**Affiliations:** ^1^ Department of Radiation Oncology, Dalin Tzu Chi Hospital, Buddhist Tzu Chi Medical Foundation, Chia-yi, Taiwan; ^2^ Cancer Centre, Dalin Tzu Chi Hospital, Buddhist Tzu Chi Medical Foundation, Chia-yi, Taiwan; ^3^ School of Medicine, Tzu Chi University, Hualien, Taiwan; ^4^ Department of Biomedical Sciences, National Chung Cheng University, Chia-Yi, Taiwan; ^5^ Georgia Cancer Center and Department of Medicine, Medical College of Georgia, Augusta University, Augusta, GA, United States; ^6^ Department of Surgical Oncology, Leiden University Medical Centre, ZA, Leiden, Netherlands

**Keywords:** radiotherapy (RT), immunotherapy, epigenetic modification, cancer, immune-oncology (IO)

Epigenetic modifications play an essential role in cancer development, disease progression/recurrence, and treatment response via regulating gene expression without changing the corresponding DNA sequence. In cancer patients treated with radiotherapy (RT) and immuno-oncology (IO), epigenetic modifications emerge as potential targets to predict and improve treatment outcomes.

RT is a common modality in treating cancers. However, cancer cells may repair their post-irradiation DNA damage, limiting RT’s clinical effectiveness. Epigenetic modifications play a critical role in DNA damage response and repair. For example, histone modifications may alter chromatin structure to change the accessibility of DNA repair machinery. Besides, DNA methylation can silence the DNA-repair genes transcriptionally, making cells more susceptible to DNA damage. Thus, investigating and targeting epigenetic modifications may enhance the clinical efficacy of RT.

Immuno-oncology (IO) is a rapidly evolving field in cancer treatment. Epigenetic modifications can regulate the expression of immune-related genes, such as immune checkpoints and cytokines. For example, DNA methylation of the promoter region of the programmed death-ligand 1 (PD-L1) gene can lead to its silencing. Thus, it is reasonable to apply epigenetic modifications as biomarkers to predict response to IO and as potential targets to enhance the treatment efficacy.

In the literature, several epigenetic modifiers, such as histone deacetylase inhibitors (HDACi) and DNA methyltransferase inhibitors (DNMTi), have shown promise in preclinical and clinical studies in the combined treatment of RT and IO. For example, HDACi can sensitize cancer cells to irradiation by inhibiting DNA repair pathways and enhancing immune-mediated tumor cell death. Six articles are presented in the present specific section, as follows.

Firstly, Wu et al. investigated the effect of endoplasmic reticulum (ER) stress on the immune system, focusing on macrophages and neutrophils in oral squamous cell carcinoma (OSCC). The authors hypothesized that ER stress could transform neutrophils into an immunosuppressive phenotype via the expression of lectin-like oxidized low-density lipoprotein receptor-1 (LOX-1). In the study, they treated two OSCC cell lines with a vehicle or an ER stress inducer, i.e., thapsigargin (THG). They collected their respective conditioned media for subsequent culturing of human peripheral blood neutrophils obtained from healthy donors. The authors found neutrophils cultured in a THG-treated conditioned medium expressed a higher level of LOX-1 and meaningfully inhibited T cell proliferation more than those cultured with a vehicle-treated conditioned medium. These novel findings suggest that ER stress in OSCC cells can relay a signal to neutrophils, making them immunosuppressive. Moreover, the results support the existence of “transmissible” ER stress between tumor cells and neutrophils.

Secondly, Cheng et al. investigated the pharmacokinetics of tamoxifen during concurrent and sequential RT in rats. The authors used ultra-high-performance liquid chromatography-tandem mass spectrometry to measure plasma tamoxifen concentration. The results indicated that tamoxifen’s area under the curve (AUC) was 1.5- to 1.7-fold higher in the RT-0.5-Gy group compared with the mock and RT-2.0-Gy groups. The relative bioavailability of tamoxifen ranged from 127% to 202% and from 71% to 152% in the concurrent RT-0.5-Gy and RT-2.0-Gy groups, respectively. The study also reported a 3- to 5-fold increase in the magnitude of endoxifen, which converted from 4-hydroxy tamoxifen and N-desmethyl tamoxifen, in the concurrent RT groups. This study confirmed the interaction between RT and hormone therapy, suggesting that RT and tamoxifen combination may potentially benefit patients with hormone-dependent breast cancer.

Thirdly, Tang et al. evaluated the predictive power of a 35-gene mutation profile and RT parameters in esophageal squamous cell carcinoma (ESCC) patients treated with definitive concurrent chemoradiotherapy (CCRT). The authors found four significant prognostic factors affecting progression-free survival, as follows: clinical nodal staging, lung volume receiving ≥30 Gy (V30), and mutation of fibrous sheath interacting protein 2 (FSIP2). For overall survival, two substantial prognostic factors were lung V30 and mutation of spectrin repeat containing nuclear envelope protein 1 (SYNE1). The Chinese patient cohort showed higher mutation rates of MUC17, FSIP2, and SYNE1 than that identified in the ESCC cohorts from The Cancer Genome Atlas. The study suggested that combining the 35-gene mutation profile and RT dosimetry could help predict the clinical outcomes of ESCC patients treated with definitive CCRT.

Fourth, Liu et al. investigated the role of NKG2D signaling in the combined treatment of RT and HDACi in the context of hepatocellular carcinoma (HCC). The authors used an *in vitro* co-culture system with NK cells and a syngeneic mouse model. The results showed that combined RT and HDACi substantially enhanced NK cell-related cytotoxicity and increased the expression of NKG2D ligands, such as MICA/MICB in human cells and RAE-1/H60 in murine cells. The delayed tumor growth *in vivo* was associated with NKG2D ligand expressions. These findings suggested potential therapeutic effects of combined RT/HDACi and NK cell-directed immunotherapy for HCC treatment.

Fifth, Hsu et al. investigated the potential benefits of Z-libutilide, a compound found in Angelica sinensis, in treating oral cancer. Previous studies demonstrated that Z-ligustilide has anti-cancer effects on various cancer types; however, its effects on oral cancer cells under hypoxic conditions were unknown. The study found that Z-ligustilide could inhibit migration of the oral cancer cell line TW2.6, resulting in caspase-dependent apoptosis under hypoxia. The authors also observed that Z-ligustilide induced c-Myc-dependent apoptosis and played a role in ER-stress signaling, suggesting a potential role of Z-ligustilide, an RT sensitizer, in inducing the death of oral cancer cells under hypoxic conditions.

Finally, Su et al. investigated the anti-cancer activity of 5-methoxy tryptophan (5-MTP) with or without Cisplatin (CDDP) in head-and-neck squamous cell carcinoma. According to their findings, the growth of SCC25 cells was decreased by CDDP, while 5-MTP did not exhibit such a substantial effect. However, when used together, CDDP and 5-MTP demonstrated an impressive inhibitory impact on the growth of SCC25 cells by reducing the phosphorylation of STAT3. There is a future potential for combining CDDP and 5-MTP with RT to treat HNSCC.

In summary, epigenetic modifications are a crucial research field in RT and IO for managing cancers. Investigating and targeting these modifications may enhance treatment efficacy and improve the clinical outcomes of patients. Although several promising results have been reported in the literature, further investigations are needed to determine the optimal use of epigenetic modifiers in combination with RT and IO.

## Author contributions

All authors listed have made a substantial, direct, and intellectual contribution to the work and approved it for publication.

